# Antimicrobial susceptibility testing of *Aggregatibacter Actinomycetemcomitans* can be performed using the EUCAST medium for anaerobic bacteria

**DOI:** 10.1080/20002297.2026.2616116

**Published:** 2026-01-19

**Authors:** Anne Birkeholm Jensen, Erika Matuschek, Gunnar Kahlmeter, Niels Nørskov-Lauritsen

**Affiliations:** a Department of Dentistry and Oral Health, Aarhus University, Aarhus, Denmark; b EUCAST Development Laboratory, Växjö, Sweden; c Department of Clinical Microbiology, Esbjerg Hospital University Hospital of Southern Denmark, Esbjerg, Denmark

**Keywords:** Disk diffusion, FAA-HB agar, azithromycin, ampicillin, periodontal pathogen, oral microbiology

## Abstract

**Background:**

The oral bacterium *Aggregatibacter actinomycetemcomitans* (*Aa*) is associated with infectious diseases treated with antibiotics, but specific recommendations for antimicrobial susceptibility testing (AST) of the species do not yet exist. Objective To evaluate the possibility to perform AST of *Aa* following EUCAST guidelines using FAA-HB and 20 h incubation.

**Method:**

Twenty-nine *Aa* strains were analysed using disk diffusion on FAA-HB media from three manufacturers. Plates with with ampicillin and azithromycin disks were incubated in 5% CO_2_ and anaerobic conditions and examined after 20 and 44 h. Strains were additionally tested with the agar dilution method, and inhibition zone diameters (IZD) were correlated to the minimal inhibitory concentrations.

**Results:**

FAA-HB agar supported sufficient growth of *Aa* after 20 h incubation in 5% CO₂. No significant differences in IZD were found between the three media tested. Prolonged incubation (44 h) increased IZD, but 20 h incubation was adequate for reproducible results. Incubation in 5% CO₂ was superior to anaerobic conditions.

**Conclusion:**

Incubation in 5% CO2 and reading after 20 h incubation resulted in the best correlation between FAA-HB media from three manufacturers. The EUCAST disk diffusion method with FAA-HB for AST of *Aa* may be crucial for future standardization and development of clinical breakpoints for this species.

## KEY MESSAGE

- Fastidious anaerobic agar with 5% horse blood reliably supports sufficient 20-h growth of *A. actinomycetemcomitans* in 5% CO₂, enabling reproducible EUCAST-compatible antimicrobial susceptibility testing.

## Introduction

is a Gram-negative, non-motile, facultative anaerobic bacterium that grows well on enriched media in ambient air supplemented with 5% CO_2_. *A. actinomycetemcomitans* appears as small, rough colonies 0.5 mm in diameter after approximately 24 h, although some strains may need 48 h for sufficient growth [1]. *A. actinomycetemcomitans* is a member of the oral microbiota but can cause severe infections when introduced in otherwise sterile compartments of the human body, e.g. brain abscess and infectious endocarditis [1–4]. *A. actinomycetemcomitans* is also associated with severe periodontitis in young individuals, currently categorised as Grade C Molar-Incisor pattern periodontitis [5–7]. Studies have reported difficulties in eliminating this bacterium from the periodontal pockets of periodontitis patients by debridement only, and treatment with antibiotics may be needed in some cases [5,8,9]. Based on clinical studies, the recommended antibiotic regimen in the treatment of periodontitis is amoxicillin plus metronidazole, which targets a broad spectrum of the oral microbiota. In the presence of *β*-lactam resistance or allergy to penicillin, azithromycin is the best choice [10].

Antimicrobial susceptibility testing (AST) of *A. actinomycetemcomitans* is important for guidance of treatment and for surveillance of antimicrobial resistance. Several studies have reported on the antimicrobial susceptibility of *A. actinomycetemcomitans*, but the studies differ in methodology, e.g. incubation (anaerobic vs. aerobic), timeframe for reading of plates, inoculum size, interpretation (breakpoints), and the media used [11–15]. Guidelines for determination of minimal inhibitory concentration (MIC) are available for the HACEK group (including *A. actinomycetemcomitans*) by CLSI (Clinical Laboratory Standard Institute), hitherto, the European Committee for Antimicrobial Susceptibility Testing (EUCAST) criteria for AST of *A. actinomycetemcomitans* have not been developed. EUCAST recommends Mueller Hinton agar supplemented with defibrinated horse blood and beta-NAD (MH-F) for disk diffusion of fastidious microorganisms [16,17], but *A. actinomycetemcomitans* grows insufficiently on MH-F with 5% horse blood for AST [12]. Also, MIC determination with broth microdilution in MH-F broth, as recommended by EUCAST for most fastidious organisms, does not support sufficient growth of *A. actinomycetemcomitans* (data not published).

Due to insufficient growth of anaerobes on MH-F agar, EUCAST investigated fastidious anaerobe agar supplemented with 5% mechanically defibrinated horse blood (FAA-HB) for AST of anaerobic bacteria [18,19], and currently recommends MIC method for anaerobic bacteria is agar dilution on FAA-HB [20,21]. The aim of this study was to evaluate if FAA-HB agar plates can support sufficient growth of *A. actinomycetemcomitans* in 20 h, which would suggest a possible use of this medium for AST of *A. actinomycetemcomitans* within mandatory EUCAST criteria. We addressed FAA-HB from three manufacturers (designated ‘biological replicates’), performed incubation in anaerobic and CO_2_-enriched atmospheres, and evaluated antimicrobial action of ampicillin and azithromycin with both disk diffusion and agar dilution.

## Materials and methods

### Study isolates and quality control strains

Twenty-nine *A. actinomycetemcomitans* strains were selected for AST (Table 1). All strains were identified to species level with MALDI-TOF [12]. The strains were subcultured in 5% CO_2_ at 35 ± 1 °C twice before being used for AST, and the strains were used for AST after 48 h of incubation. *A. actinomycetemcomitans* (ATCC 43718), *Clostridium perfringens* (ATCC 13124), and *Bacteroides fragilis* (ATCC 25285) were used for quality control.

**Table 1.** Study strain collection.

Isolate	Origin	Reference (year)
ATCC 33384^T^	Oral abscess	Klinger (1912)
ATCC 43718/HK 975/Y4	Subgingival plaque	Klinger (1912)
ATCC700685/CCUG 56173/HK 1651	Periodontitis	J. Westergård, Denmark (1996)
CCUG 51668/HK 929	Dental plaque	S. Asikainen, Finland (before 1992)
CCUG 51667/HK 928	Dental plaque	S. Asikainen, Finland (before 1992)
CCUG 56172/HK 921/JP2	Periodontitis	C.C. Tsai, USA (1984)
C100	Unknown	R. Clasesson, Sweden
C10000	Unknown	R. Claesson, Sweden
D7ss	Oral isolates	Y. Wang, USA (-)
HK 907	Periodontitis	T.M.J van Steenbergen, the Netherlands
HK 909, HG1080	Unknown	Denmark (1990s)
HK 1613	Periodontitis	DiRienzo, USA (1990s)
HK 1615	Periodontitis	DiRienzo, USA (1990s)
PN604	Periodontitis	A.J. van Winkelhoff, the Netherlands (2000s)
PN603	Periodontitis	A.J. van Winkelhoff, the Netherlands (2000s)
PN385	Unknown	N. Nørskov-Lauritsen, Denmark (-)
RRI 700 541/UK11	Periodontitis	D. Ready, UK (before 2017)
RRI 700 542/UK7	Periodontitis	D. Ready, UK (before 2017)
RRI 700 543/UK42	Periodontitis	D. Ready, UK (before 2017)
RRI 700 544/UK21	Periodontitis	D. Ready, UK (before 2017)
RRI 700 545/UK44	Periodontitis	D. Ready, UK (before 2017)
RRI 700 546/UK48	Periodontitis	D. Ready, UK (before 2017)
RRI 700 547/UK4	Periodontitis	D. Ready, UK (before 2017)
RRI 700 548/UK46	Periodontitis	D. Ready, UK (before 2017)
RRI 700 549/UK14	Periodontitis	D. Ready, UK (before 2017)
RRI 700 550/UK1	Periodontitis	D. Ready, UK (before 2017)
RRI 700 551/UK17	Periodontitis	D. Ready, UK (before 2017)
RRI 700 552/UK12	Periodontitis	D. Ready, UK (before 2017)
524 G	Oral isolate	D. Haubek/A. Johansson, Ghana (2009)

### AST by disk diffusion

The disk diffusion method was carried out according to EUCAST guidelines and the 15-15-15 rule [16,22] on in-house prepared FAA-HB using FAA from three manufacturers (Bioconnections (BioConnections, Brindley Court Knypersley, UK), Neogen (NEOGEN, Heywood, BL9 7JJ, UK), and EO Labs (E&O Laboratories Ltd., Burnhouse, Bonnybridge, Scotland). Antibiotic disks with 2 µg ampicillin (Oxoid/Thermo Fischer Scientific, Basingstoke, UK) and 15 µg azithromycin (Oxoid/Thermo Fischer Scientific, Basingstoke, UK) were tested. Plates were incubated in 5% CO_2_ at 35 ± 1 °C and anaerobically (80% N_2_, 10% CO_2_, 10% H_2_) (Whitley A35 anaerobic workstation, West Yorkshire, UK) at 35 ± 1 °C and visually inspected after 16–20 h and after 40–44 h. The quality (paleness and richness of growth) and confluence of growth, the appearance of zone edges, and the ease with which the zone diameter could be measured were evaluated according to EUCAST criteria [23]. The tests were performed in triplicate.

### MIC determination by the agar dilution method

Stock solutions of ampicillin and azithromycin (European Pharmacopoiea Reference Standard, Sigma-Aldrich, Germany) were prepared at a concentration of 1000 mg/L. Ampicillin stock solution was prepared in 0.1 M phosphate buffer, pH 8.0, and azithromycin in 95% ethanol [20,24]. Agar dilution plates were prepared as described by EUCAST [20]. Briefly, medium for FAA-HB plates (E&O Laboratories Ltd., Burnhouse, Bonnybridge, Scotland) was supplemented with 5% defibrinated horse blood and various concentrations of antibiotics. McFarland suspensions of 0.5 were prepared in sterile saline (0.9%) from plates with bacteria cultured for 48 h. The suspensions were diluted 10 times, equivalent to a concentration of 10^7^ colony-forming units (CFU) per ml. One microliter (approximately 10^4^ CFU) was inoculated onto the agar plates using a Denley Multipoint Inoculator.

Each assessment was composed of nine plates with two-fold dilutions of antibiotic (range 0.0625–16.0 mg/L) plus two control plates without added antibiotic. Plates were incubated in 5% CO_2_ at 35 ± 1 °C and visually inspected after 20 h. MIC values were defined as the lowest concentrations of the antimicrobial agent that inhibited visible growth, disregarding a single colony or a thin haze at the inoculated spot (EUCAST & ESCMID, 2000). All tests were performed in triplicate, and the median MIC were used for further analysis.

### Data analysis

Data analysis was performed using Excel and GraphPad Prisms 10.4.0 (621). Media comparison under the different incubation forms and interval was done by use of a two-way ANOVA. The difference in mean inhibition zone diameter from the different incubation forms and intervals was calculated using the Wilcoxon matched pairs signed rank test for non-parametric data and a non-paired t-test for parametric data. The correlation between inhibition zone diameter and MIC was calculated by use of a simple linear regression model.

## Results

### Disk diffusion

**Figure 1.**
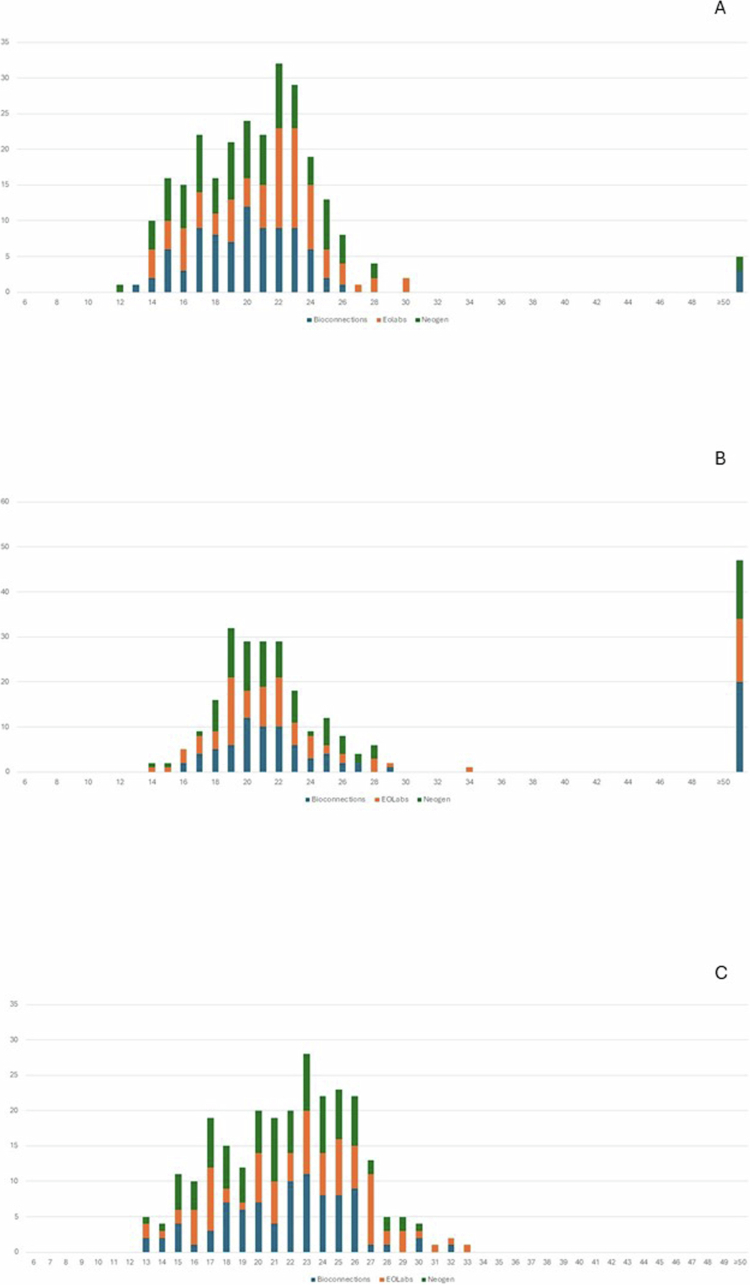
Inhibition zone diameters for ampicillin and *A. actinomycetemcomitans* on FAA-HB from Bioconnection, Neogen and EOLabs (261 readings). A) 5% CO_2_, 20 h incubation B) Anaerobic chamber, 20 h incubation C) 5% CO_2_, 44 h incubation. Number of isolates (y-axis) with a certain inhibition zone diameter (x-axis) obtained on media prepared from FAA from three different manufacturers. No significant difference was observed among the FAA-HB manufacturers. The different FAA media are represented by colours. Blue columns: Bionnections; orange columns: EOLabs; green columns: Neogen. The last column to the right shows strains showing no growth (failure).

**Figure 2.**
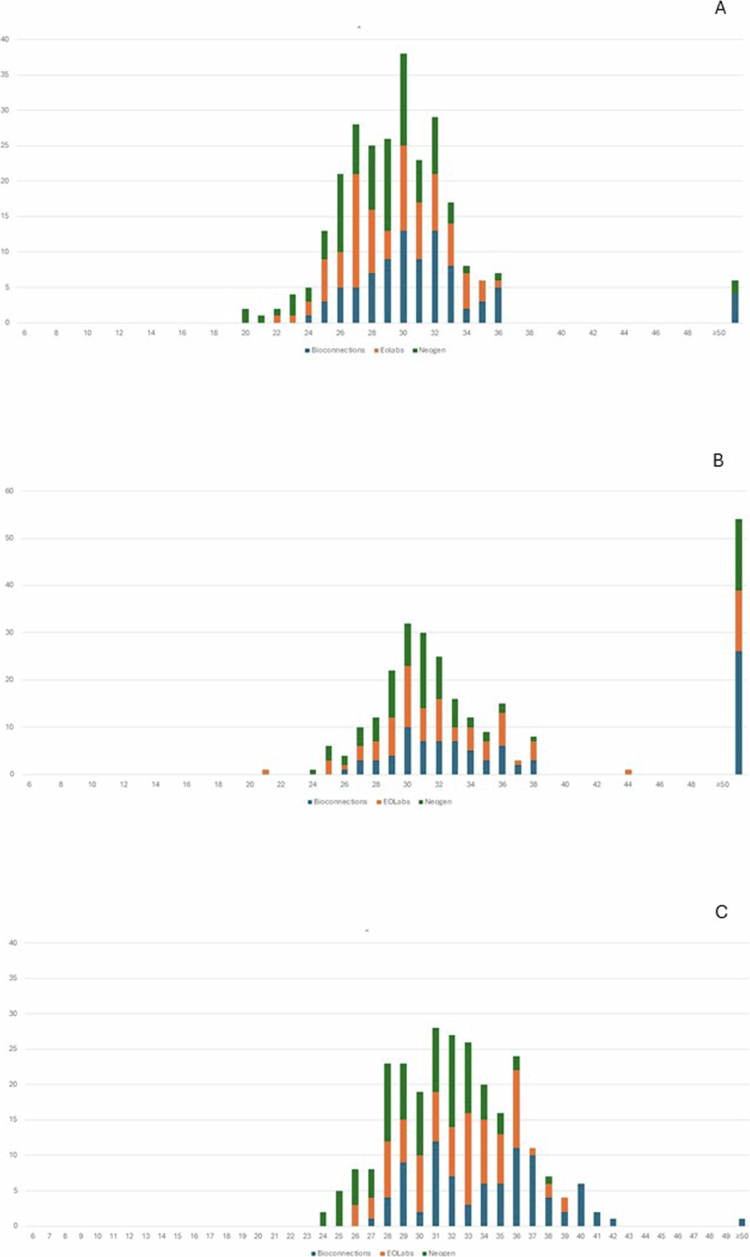
Inhibition zone diameters for azithromycin and *A. actinomycetemcomitans* on FAA-HB from Bioconnection, Neogen and EOLabs (261 readings). A) 5% CO_2_, 20 h incubation B) Anaerobic chamber, 20 h incubation C) 5% CO_2_, 44 h incubation. Number of isolates (y-axis) with a certain inhibition zone diameter (x-axis) obtained on media prepared with FAA from three different manufacturers. No significant difference was observed among the FAA-HB manufacturers. The different FAA media are represented by colours. Blue columns: Bionnections; orange columns: EOLabs; green columns: Neogen. The last column to the right shows strains showing no growth (failure).

FAA-HB media from the three manufacturers resulted in confluent growth for 259 of 261 measurements (99%) of the 29 study strains after 20 h incubation in 5% CO_2_, and for all investigated organisms after 44 h in 5% CO_2_ (Table S1). Under anaerobic incubation, 235 (90%) measurements showed confluent growth after 20 h and 253 (97%) after 44 h. The mean difference of the inhibition zone diameter obtained on the three media was not statistically significant (*p* > 0.05), and the zone diameters were similar (Figures 1 and 2 and Table 2). The difference in mean inhibition zone diameters obtained on FAA-HB from three manufactures were under 2 mm, except for Bioconnection and Neogen on the azithromycin disk where the difference reached 2.1 mm (Table 2). By visual inspection of Figures 1 and 2, media from EOlabs performed superior to the other two media after 20 h incubation in 5% CO_2_, since all strains exhibited visible growth. The media from Bioconnection resulted in more growth failures after 20 h in anaerobic incubation (Table S1). However, the difference between the media was not statistically significant (*p* > 0.05).

**Table 2.** Mean inhibition zone diameter of the ampicillin and azithromycin disk determined on FAA from three different manufactures (20 h incubation in 5% CO_2_).

Antibiotic disk	Bioconnection mean mm (sd)	Neogen mean mm (sd)	EO Labs mean mm (sd)
Ampicillin	19.8 (3)	20.1 (3.8)	21.1 (3.5)
Azithromycin	30.4 (2.9)	28.3 (3.1)	29.3 (3.2)

Overall, the strains grew sufficiently after 20 h of incubation in 5% CO_2_ for reproducible interpretation of inhibition zone diameters (Table S1). More specifically, all strains showed confluent growth on EOLabs, whereas one strain resulted in semi-confluent growth on Bioconnection and Neogen. Under anaerobic incubation, a considerable number of strains needed prolonged incubation for 44 h to reach confluent growth (Figures 1B–2B and Table S1). Prolonged incubation for 44 h in 5% CO_2_ resulted in confluent growth of all strains, but with a statistically significant larger mean inhibition zone diameters compared to incubation for 20 h only (*p* < 0.05) (Figures 1C–2C and Table 3). Prolonged incubation in the anaerobic chamber also showed a tendency of increased inhibition zone diameters, especially for azithromycin (Table 3).

**Table 3.** Mean inhibition zone diameter of the ampicillin and azithromycin disk after incubation in 5% CO_2_ and anaerobic chamber for 20 and 44 h.

Antibiotic disk	5% CO_2_, 20 h mean mm (sd)	5% CO_2_, 44 h mean mm (sd)	Anaerobic, 20 h mean mm (sd)	Anaerobic, 44 h mean mm (sd)
Ampicillin	20.32 (3.5)	21.9 (4.2)	21.3 (3.3)	23.5 (3.8)
Azithromycin	29.3 (3.2)	32.3 (4.3)	32.3 (12.3)	34.8 (4.7)

The quality of the growth, determined by the confluence and richness of the growth and the number of strains readable after 20 h, was superior with incubation in 5% CO_2_compared to incubation in anaerobic chamber (Table S2).

Based on the overall summary of the results, incubation in 5% CO_2_ and reading after 20 h resulted in the best correlation between the three different media.

### Correlation between inhibition zone diameters and MICs

**Figure 3.**
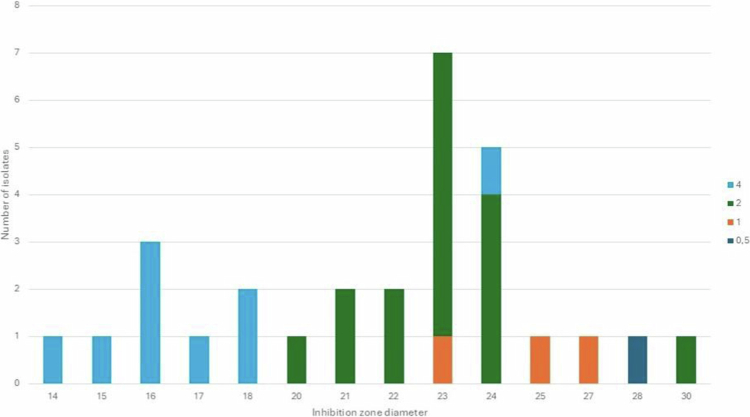
Correlation of inhibition zone diameters for ampicillin and MICs. Inhibition zone diameter distribution for *A. actinomycetemcomitans* and ampicillin 2 µg with corresponding MIC values as coloured bars. Results from incubation in 5% CO_2_ for 20 h. Number of isolates (y-axis) with an inhibition zone diameter (x axis; mm) and correlating MIC (colour-coded columns). The correlation observed between the MIC and inhibition zone diameters was acceptable. Blue columns: isolates with an MIC of 4 mg/L; green columns: isolates with an MIC of 2 mg/L; orange columns: isolates with an MIC of 1 mg/L; dark blue columns: isolates with an MIC of 0.5 mg/L.

Figure 3 shows the distribution of MIC in relation to the correlating inhibition zone diameter. Based on the linear regression analysis, the correlation between the MIC and inhibition zone diameters with ampicillin and 20 h incubation in 5% CO_2_ was acceptable demonstrated by a r^2^ of 0.66 (*p* < 0.001). For the azithromycin disk, the correlation was poor with an r^2^ 0.36 (*p* < 0.0056). With some exceptions, Figure 3 shows that strains determined with an MIC of 4 mg/L were more likely to have smaller inhibition zone diameters, and strains determined with an MIC of 1 or 0.5 mg/L had inhibition zone diameters above 22 mm. More specifically, eight of nine isolates with a MIC of 4 mg/L exhibited zones ≤ 19 mm, while all isolates with a MIC ≤ 2 mg/L exhibited zones > 19 mm.

## Discussion

We have demonstrated that the anaerobic FAA-HB agar is useful for AST with the disk diffusion method of *A. actinomycetemcomitans* and incubation in 5% CO_2_. Only 5 of 261 (2%) measurements failed to sustain sufficient growth for visible evaluation, in contrast to 18% failures after anaerobic incubation on the same media. This medium consistently yielded confluent growth with similar inhibition zone diameters after 20 h incubation in air with 5% CO_2_, providing a robust and dependable method for AST of *A. actinomycetemcomitans*.

The strains showed excellent growth on FAA-HB from three manufacturers, and the difference in inhibition zone diameters obtained with the different FAA-HB was acceptable because the mean difference in inhibition zone diameters were two millimetres or less and did not reach statistical significance. The capnophilic species, *A. actinomycetemcomitans*, grows well in air with 5% CO_2_, but we additionally tested the species with anaerobic incubation because FAA-HB is a media developed for testing of anaerobic species and performs well under anaerobic conditions [18].

The results obtained for this species, and the disk diffusion method were most promising with the use of ampicillin compared to azithromycin. Clinical studies recommend the use of amoxicillin and metronidazole for the treatment of *A. actinomycetemcomitans*-associated periodontitis, and the acceptable correlation between the MIC and the inhibition zone diameter on the ampicillin disk makes our results promising for future treatment planning [10].

We need additional data to establish epidemiological cut-off values (ECOFFs) for *A. actinomycetemcomitans*, which will be an important part of the breakpoint setting procedure by EUCAST [25]. ECOFFs apply to individual species only. Applying ECOFFs or clinical breakpoints for systemic pathogens to oral species may yield unreliable antibiotic susceptibility interpretations. Therefore, developing ECOFFs or clinical breakpoints for relevant oral species will improve antibiotic selection for the treatment of oral infections. Our strain population showed a reasonable distribution of inhibition zone diameters and MICs, and considering *Aggregatibacter* to be resistant to ampicillin goes against clinical guidelines and empirical use of ampicillin/amoxicillin in the treatment of *Aggregatibacter*-associated diseases [10]. Previously, 27 of the 29 study strains examined in the present study were categorised with a one two-fold dilution step lower amoxicillin MICs using blood agar and 44 h incubation. Precisely the media and incubation time may be a plausible explanation for the difference between the results. However, our results emphasise the importance of following the criteria and guidelines provided by, e.g. EUCAST, to get reliable results, and the correlation between inhibition zone diameter and MIC should be tested on a larger strain collection.

Previous studies have reported difficulties with haze zones within the inhibition zones [12]. In the present study, we did not experience such haze zones, and overall, the inhibition zone diameters were easy to interpret with clear edges. However, the recommendation by EUCAST concerning the handling of plates, e.g. using over-night dried plates with room temperature, was strictly followed in the present study, which may have eliminated haze zones due to humidity.

Some limitations of the present study should be acknowledged. We cannot categorise any of the study strains as resistant or susceptible due to the lack of clinical breakpoint for this species. In addition, the small strain collection calls for future studies testing the method on a larger strain collection that includes resistant strains and additional antimicrobial agents.

The disk diffusion method is considered a reproducible and sustainable method for AST [16]. Standardising the method for *A. actinomycetemcomitans* would improve the differentiation between wild-type strains (strains without phenotypically detectable resistance mechanisms) and non-wild-type strains (strains with phenotypically detectable resistance mechanisms) within this species in clinical settings [26]. There is a need for reproducible, easy, and available methods for AST of oral pathogens that cause disease both intra- and extra-orally [27]. Standardised AST of oral strains will ensure reproducible and comparable results that clinically benefit patients, clinicians, and society both concerning treatment planning and surveillance of the development of antimicrobial resistant in members of the oral microbiota. The lack of reproducibility of previous published results emphasises the importance of establishing criteria and guidelines for AST of oral pathogens like *A. actinomycetemcomitans*. The EUCAST disk diffusion method and FAA-HB is feasible for this purpose, and the results of the present study prepare the ground for further standardising the disk diffusion method for *A. actinomycetemcomitans* and encourage future studies aiming at developing clinical breakpoints.

## Supplementary Material

Table_Suppl.docx Supplemental Material
