# Supplementary material for: Antimicrobial susceptibility testing of Aggregatibacter Actinomycetemcomitans can be performed using the EUCAST medium for anaerobic bacteria
Source: J Oral Microbiol. 2026 Jan 19;18(1):2616116. doi: 10.1080/20002297.2026.2616116 (PMC12818291; doi:10.1080/20002297.2026.2616116)
Supplement: Table_Suppl.docx — Supplemental Material [file ZJOM_A_2616116_SM9611.docx]

**Table S1.** Quality of growth of triplicated measurements of 29 study strains determined for FAA-HB from three different manufactures.

| Growth | Bioconnection | | | | Neogen | | | | EOLabs | | | |
| --- | --- | --- | --- | --- | --- | --- | --- | --- | --- | --- | --- | --- |
| Atmosphere  Hr | CO_2_  20 | CO_2_  44 | An  20 | An  44 | CO_2_  20 | CO_2_  44 | An  20 | An  44 | CO_2_  20 | CO_2_  44 | An  20 | An  44 |
| Confluent (n^*^) | 86 | 87 | 75 | 83 | 86 | 87 | 80 | 86 | 87 | 87 | 80 | 84 |
| Semi-confluent (n) | 1 | 0 | 1 | 1 | 1 | 0 | 1 | 1 | 0 | 0 | 1 | 2 |
| Pale (n) | 33 | 4 | 51 | 39 | 14 | 3 | 48 | 38 | 5 | 84 | 44 | 29 |
| Rich (n) | 54 | 83 | 25 | 45 | 73 | 84 | 33 | 49 | 82 | 0 | 37 | 57 |
| No growth (n) | 0 | 0 | 11 | 3 | 0 | 0 | 6 | 0 | 0 | 0 | 6 | 1 |

| Growth | 5 % CO_2_, 20 hr | 5 % CO_2_, 44 hr | Anaerobic, 20 hr | Anaerobic, 44 hr |
| --- | --- | --- | --- | --- |
| Confluent (n^*^) | 259 | 261 | 235 | 253 |
| Semi-confluent (n) | 2 | 0 | 3 | 4 |
| Pale (n) | 52 | 7 | 92 | 105 |
| Rich (n) | 209 | 254 | 143 | 151 |
| No growth (n) | 0 | 0 | 23 | 4 |

**^*^**Number of strains

**Table S2.** Quality of growth of triplicated measurements of 29 study strains determined after incubation in 5 % CO_2_ and anaerobic chamber for 20 and 44 hours.

**^*^**Number of strains
